# Living on the edge: conservation genetics of seven thermophilous plant species in a high Arctic archipelago

**DOI:** 10.1093/aobpla/plx001

**Published:** 2017-01-19

**Authors:** Siri Birkeland, Idunn Elisabeth Borgen Skjetne, Anne Krag Brysting, Reidar Elven, Inger Greve Alsos

**Affiliations:** 1 The University Centre in Svalbard, P.O. Box 156, Longyearbyen, NO 9171, Norway; 2 Centre for Ecological and Evolutionary Synthesis, Department of Biosciences, University of Oslo, P.O. Box 1066 Blindern, Oslo, NO 0316, Norway; 3 Natural History Museum, University of Oslo, P.O. Box 1172 Blindern, Oslo, NO 0318, Norway; 4 Tromsø University Museum, UiT The Arctic University of Norway, P.O. Box 6050 Langnes, Tromsø, NO 9037, Norway

**Keywords:** Amplified fragment length polymorphism (AFLP), Arctic, climate change, conservation genetics, edge populations, islands, regional red list, Svalbard

## Abstract

Small, isolated and/or peripheral populations are expected to harbour low levels of genetic variation and may therefore have reduced adaptability to environmental change, including climate warming. In the Arctic, global warming has already caused vegetation change across the region and is acting as a significant stressor on Arctic biodiversity. Many of the rare plants in the Arctic are relicts from early Holocene warm periods, but their ability to benefit from the current warming is dependent on the viability of their populations. We therefore examined Amplified Fragment Length Polymorphism (AFLP) data from regional red listed vascular plant species in the High Arctic archipelago of Svalbard and reference populations from the main distribution area of: (1) *Botrychium lunaria*, (2) *Carex capillaris* ssp. *fuscidula*, (3) *Comastoma tenellum*, (4) *Kobresia simpliciuscula* ssp. *subholarctica*, (5) *Ranunculus wilanderi*, (6) *Sibbaldia procumbens* and (7) *Tofieldia pusilla*. In addition, we gathered population size data in Svalbard. The Svalbard populations had low genetic diversity and distinctiveness and few or no private markers compared to populations outside the archipelago. This is similar to observations in other rare species in Svalbard and the genetic depletion may be due to an initial founder effect and/or a genetic bottleneck caused by late Holocene cooling. There seems to be limited gene flow from other areas and the Svalbard populations should therefore be considered as demographically independent management units. Overall, these management units have small and/or few populations and are therefore prone to stochastic events which may further increase vulnerability to inbreeding depression, loss of genetic variation, and reduced evolutionary potential. Our results support theory predicting lower levels of genetic diversity in small, isolated and/or peripheral populations and may be of importance for management of other rare plant species in the Arctic.

## Teaser

Small and peripheral populations often contain low levels of genetic variation. This may limit their ability to adapt to environmental change, including climate warming. In a recent study published in AoB PLANTS, Birkeland, Skjetne and colleagues show that many rare and threatened plant species in the High Arctic archipelago Svalbard harbour low levels of genetic variation. Most of them are probably relicts from the early Holocene warmer period. They have likely experienced strong genetic founder/bottleneck effects due to climatic limitations. Even though temperatures now are rising, it is highly uncertain whether this will be beneficial for these warmth-demanding species.

## Introduction

Small, isolated and/or peripheral populations may harbour low levels of genetic variation due to genetic drift, inbreeding, bottlenecks and founder effects (Ellstrand and Elam 1993; Frankham 1996; Cole 2003; Frankham *et al.* 2010). For island populations, reduction of genetic variation is expected to be greater the lower the number of founders, the smaller the population sizes, the lower the immigration rates, the smaller the island size, and the greater the distance to the mainland (Jaenike 1973; Frankham 1997). Similarly, the central–marginal hypothesis also predicts a decline in within-population genetic diversity and increase in genetic differentiation towards range margins, although observed differences from empirical studies are generally small and not consistent (Gaston 2003; Eckert *et al.* 2008; Hardie and Hutchings 2010). Small, isolated and/or peripheral populations are therefore expected to have reduced adaptability to environmental change (Frankham 1997; Frankham 2005). Low levels of genetic variation also make such populations susceptible to genetic threats like inbreeding depressions and further loss of genetic variation through genetic drift, which can interact with environmental stressors and increase extinction risk (Frankham 1997). Thus, levels of genetic variation are key information when trying to understand and predict the response of small, isolated and/or peripheral populations to future environmental change. Genetic data may also give valuable information about species history (e.g. population fragmentation, bottlenecks, refugia and range shifts; Young *et al.* 1996; Petit *et al.* 2003; Meirmans *et al.* 2011), and is also essential for delineating conservation units like evolutionarily significant units (ESUs) and management units (MUs) (Moritz 1994; Sherwin and Moritz 2000; Funk *et al.* 2012). An ESU can be defined as one or several populations that are especially important for maintaining the evolutionary potential of a species due to high genetic and ecological distinctiveness (Moritz 1994; Sherwin and Moritz 2000; Funk *et al.* 2012). At a lower level, an ESU is often built up of demographically independent populations called management units, which, in contrast to ESUs, can be delineated solely on the basis of neutral markers (Funk *et al.* 2012). Management units are important for the long-term persistence of the species and are often useful for short-term management goals like monitoring habitat and population status (Funk *et al.* 2012).

The Arctic has been warming at approximately twice the global rate since the 1980s (Anisimov *et al.* 2007), and we are now experiencing vegetation change across the region (Larsen *et al.* 2014) seen as phenology changes (Menzel *et al.* 2006; Ovaskainen *et al.* 2013; Zeng *et al.* 2013), increased photosynthetic activity (Xu et al. 2013), and species shifting their ranges towards higher latitudes (Parmesan and Yohe 2003; Root *et al.* 2003; Chen *et al.* 2011). The rapid rise in temperature is expected to continue throughout the century (IPCC 2013), and the question is how Arctic ecosystems will respond to this climate change. In this context, Arctic islands may provide important study systems and sentinels. Island populations have a much higher risk of extinction than mainland populations, and the possibility of range displacement may be limited (Frankham 1997). This regards especially species which are already rare and thus more prone to stochastic events (genetic, demographic and environmental stochasticity as well as random catastrophes, Shaffer 1981; Lande 1988; Lande 1993). Increased knowledge on such species may help to make more effective decisions for biodiversity conservation.

The remote High Arctic archipelago Svalbard (74–81°N and 10–35°E) is among the best studied regions in the Arctic, with detailed knowledge of the local distribution of species (Elven *et al.* 2011; Alsos *et al.* 2016a). About one fourth of the 184 native vascular plant species in Svalbard are on the regional red list (Henriksen and Hilmo 2015), and many of these are relatively warmth-demanding compared to the more common plant species (Engelskjøn *et al.* 2003; Elven *et al.* 2011; Henriksen and Hilmo 2015; Alsos *et al.* 2016a). It is believed that the thermophilous (i.e. warmth-loving) species of Svalbard might be relicts of larger populations established between 9000 and 4000 years ago (Alsos *et al.* 2002; Engelskjøn *et al.* 2003; Alsos *et al.* 2007; Gussarova *et al.* 2012), as an early Holocene warm period is well documented in a number of proxy records from the Svalbard and western Barents Sea region (Birks 1991; Birks *et al.* 1994; Hald *et al.* 2004; Alsos *et al.* 2016b). However, for species with only one or a few populations, more recent dispersal might be just as likely (Gussarova *et al.* 2012). Despite its remote location, long distance dispersal to Svalbard has been frequent (Alsos *et al.* 2007, 2015), but restricted seed production, especially in the thermophilous species, limits dispersal within the archipelago today (Cooper *et al.* 2004; Alsos *et al.* 2007, 2013). As the temperature rises, it could be anticipated that warmth-demanding species will become increasingly common, and cold-adapted species will become increasingly rare. However, an increase in temperature might come with several additional changes like reduced snow cover and thawing of permafrost (McBean *et al.* 2005). The loss of snow cover will not only expose plants to harmful sub-zero ambient temperatures and large temperature fluctuations, but may also lead to damage by winter desiccation, repeated freeze–thaw cycles and abrasion by windblown ice particles (Walker *et al.* 1999). We therefore believe that population size data on the rare and warmth-demanding plant species on Svalbard may prove valuable in monitoring ecosystem change. In addition, the warmth-demanding plant species may turn out to play an important role in ecosystem adaptation, but this will depend on the genetic state of the populations (i.e. that they are not too genetic depauperate and subject to inbreeding depression) as well as other ecological requirements and competitive abilities (Walker 1995; Callaghan *et al.* 2005; Crawford 2008).

In this study, we gather population size data and examine Amplified Fragment Length Polymorphism (AFLP) data from several red listed vascular plant species in Svalbard. Based on the regional red list from 2006 (Kålås *et al.* 2006; [**see Supporting Information—Table S1**]), seven study species were chosen as they all were in need of more data to ensure informed conservation decisions. Our aim is to (i) evaluate their vulnerability in terms of population size and genetic diversity in Svalbard, (ii) examine their genetic relationships to populations outside Svalbard and (iii) determine if the Svalbard populations constitute management units with special conservation value.

## Methods

### Study species

**Figure 1:**
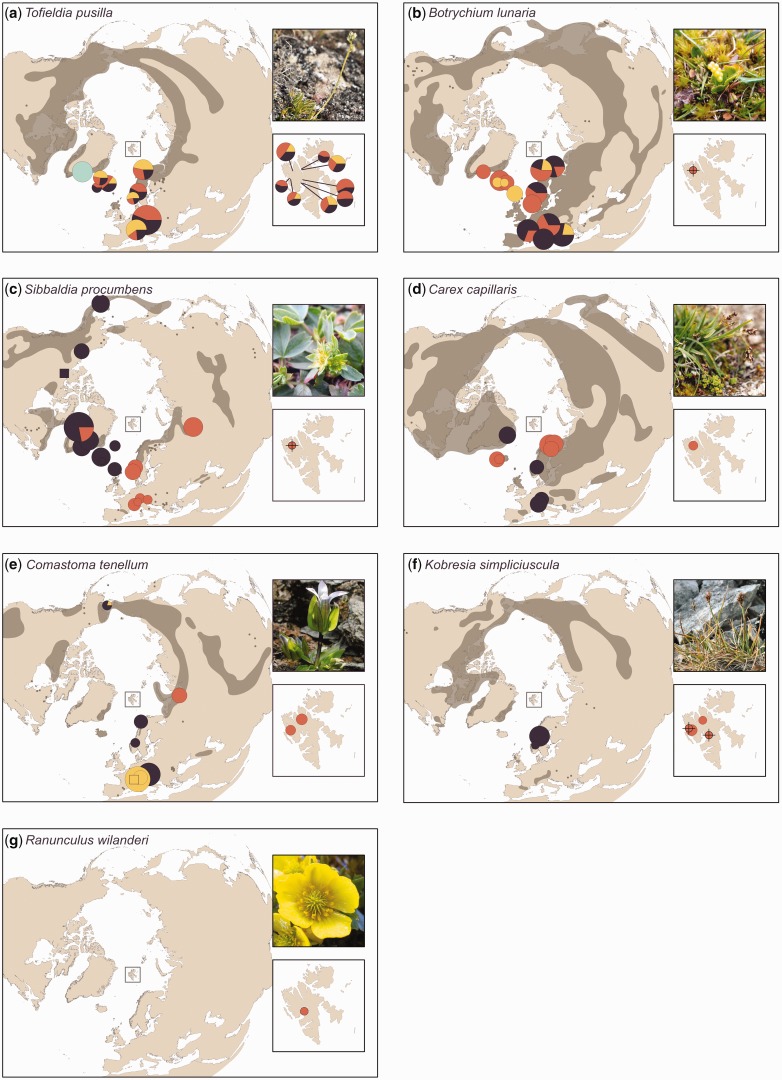
Distribution maps showing main STRUCTURE groups and genetic diversity in the seven study species. The pie charts represent the genetic groups delineated by STRUCTURE (averages over multiple runs) and the size of each pie chart is proportional to the *D* value of each population. *D* values of zero are indicated with a cross and squares are used instead of circles for populations with too small sample size to calculate genetic diversity. Species distributions are drawn after the maps of Hultén and Fries (1986) and are indicated in transparent dark grey (larger areas and small dots). (a) *Tofieldia pusilla*, (b) *Botrychium lunaria*, (c) *Sibbaldia procumbens*, (d) *Carex capillaris* (both subspecies), (e) *Comastoma tenellum*, (f) *Kobresia simpliciuscula* (both subspecies) and (g) *Ranunculus wilanderi*. Photos: Ólüf Birna Magnúsdóttir, Inger Greve Alsos (Alsos *et al.* 2016a) and Siri Birkeland.

The seven study species are: (1) *Botrychium lunaria*, (2) *Carex capillaris* ssp. *fuscidula*, (3) *Comastoma tenellum*, (4) *Kobresia simpliciuscula* ssp. *subholarctica*, (5) *Ranunculus wilanderi*, (6) *Sibbaldia procumbens* and (7) *Tofieldia pusilla* (Fig. 1; **[see Supporting Information—Tables S1 and S2]**). All species are seed plants, except *B. lunaria*, which is a pteridophyte. Furthermore, all are herbaceous plants with larger distributions outside Svalbard. However, *R. wilanderi* is considered an endemic microspecies for the archipelago (Elven *et al.* 2011). Most species are diploid [**see Supporting Information—Table S2**], and are therefore not expected to harbour hidden genetic variation in the form of fixed heterozygosity which is so common in many Arctic plants (Brochmann and Steen 1999; Brochmann and Brysting 2008).

### Population size estimation in Svalbard

To estimate population sizes in Svalbard, we either counted all visible individuals, or extrapolated the total population size from the number of individuals counted in a smaller area. Tussocks or clusters of clonal plants were treated as single individuals if they were separated by more than five centimetres, although we cannot be entirely sure that they were not connected belowground. All previously recorded localities for the seven species were revisited (Table 1). In addition, we searched for the plants in areas that could provide suitable habitat (within bioclimatic subzone C, the Middle Arctic Tundra Zone; Elvebakk 2005; Walker *et al.* 2005).

**Table 1.** Population sizes for the seven study species.

Species	Locality	Pop. ID	Date visited*	Distribution area (m^2^)	No. of individuals	Fertile	Comments
*Botrychium lunaria*	Bockfjorden, Haakon VII Land	Bl01	2/8-2009	33	21	Yes	
				**Sum:**	**21**		
*Carex capillaris* ssp. *fuscidula*	Bockfjorden, Haakon VII Land	Cc01	1/8-2009	60,000	>2000	Yes	
				**Sum:**	**>2000**		
*Comastoma tenellum*	Ossian Sarsfjellet, Haakon VII Land	Ct01	20/7-2009	51	171	Yes	
	Flatøyrdalen, Ny-Fries Land,	Ct03	24/8-2010	300	300-400	Yes	
	Ringhorndalen, Ny-Fries Land	Ct04	25/8-2010	20	>50	Yes	Population discovered for the first time by authors of the present study.
				**Sum:**	**ca. 550-650**		
*Kobresia simpliciuscula* ssp. *subholarctica*	Gipsvika, Bünsow Land	Ks01	5/7-2009	246	20**	No	
	Adolfbukta, Dickson Land/Bünsow Land	–	Elvebakk (1993)		«small»	–	
	Mimerdalen, Dickson Land	–	1925	–	–	–	Not found since 1925 (Henriksen and Hilmo 2015).
	Ossian Sarsfjellet, Haakon VII Land	Ks02	19/7-2009	800	60**	No	
	Blomstrandøya, Haakon VII Land	Ks03	19/7-2009	200	14**	Yes	
	Flatøyrdalen A, Ny Friesland	Ks04	24/7-2010	75	9**	?	Probably not corresponding to the Flatøyrdalen population discovered by Elvebakk and Nilsen in 2002 (Flatøyrdalen B).
	Flatøyrdalen B, Ny Friesland	–	Elvebakk and Nilsen (2002)	–	5	–	
	Reinsbukkdalen, Ny Friesland	–	Elvebakk and Nilsen (2002)	–	10–20	–	
	Lemströmfjellet A, Ny Friesland	–	10/7-2011	1	2–3*	?	These individuals were found South-East of Austbotnhytta and might not correspond to the 50 individuals found by Elvebakk and Nilsen in 2002 (Lemströmfjellet B).
	Lemströmfjellet B, Ny Friesland	–	Elvebakk and Nilsen (2002)	225	50	–	
				**Sum:**	**> 115**		
*Ranunculus wilanderi*	Kapp Thordsen, Dickson land	Rw01	18/7-2009	2000	51	Yes	
				**Sum:**	**51**		
*Sibbaldia procumbens*	Bockfjorden, Haakon VII Land	Sp01	1/8-2009	3000	>1000	Yes	Mature seeds observed on voucher.
		Sp02					
		Sp03					
*Tofieldia pusilla*	Blomesletta, Dickson Land	Tp01	6/7-2009	600	51	Yes	
	Kapp Nathorst, Dickson Land	Tp04	24/7-2009	403	ca. 1000	Yes	Population discovered for the first time by authors of the present study.
	Kapp Wijk, Dickson Land	Tp03	24/7-2009	800	>146	Yes	
	Blomstrandøya, Haakon VII Land	Tp02	19/7-2009	63	24	Yes	
		Tp02	19/7-2009	72	33	Yes	
	Bockfjorden, Haakon VII Land	Tp05	1/8-2009	6	100	Yes	
		Tp05	2/8-2009	1	6	Yes	
	Ossian Sarsfjellet, Haakon VII Land	Tp18	5/8-2010	3	5	Yes	
	Flatøyrdalen, Ny-Fries Land	Tp16	24/8-2010	300	9	Yes	
	Ringhorndalen, Ny-Fries Land	Tp17	25/8-2010	1000	100	Yes	Population discovered for the first time by authors of the present study.
				**Sum:**	**ca. 1500**		

Headings: Species and, if applicable, subspecies name [Species]; name of locality and region in Svalbard [Locality]; population ID used in genetic analyses (Table 2) [Pop. ID]; date for population inspection [Date visited]; extent of area where the species occurred [Distribution area (m^2^)]; counted/estimated number of individuals/ramets [No. of individuals]; whether fertile individuals were present at the time of the visit (yes/no) [Fertile].

* The populations were visited as part of the present study unless otherwise is stated.

** Tussocks.

### Plant material

Plant material for AFLP fingerprinting was collected from most visited Svalbard localities (Table 2). In addition, reference material was sampled from other Arctic-alpine populations within the species' distribution ranges (Table 2). However, for *Kobresia simpliciuscula* ssp. *subholarctica* we were only able to obtain material from a different subspecies, the European ssp. *simpliciuscula* (Elven *et al.* 2011). Also note that material from two assumed subspecies is included for *Carex capillaris*: ssp. *fuscidula* and ssp. *capillaris* (Table 2). The Svalbard population is believed to belong to the circumpolar-alpine ssp. *fuscidula* (Elven *et al.* 2011). From each Svalbard population and each reference population, fresh and healthy leaves from (if possible) ten plants were collected 2–10 m apart, and immediately stored in silica gel. A closely related species (two for *Tofieldia pusilla*) was also sampled for all study species to serve as outgroup in the neighbour-joining analysis (see below, Table 2). Herbarium vouchers from most populations are deposited in the herbariums at the University of Oslo (O) and the University of Tromsø (TROM). Plant material and AFLP data for *Sibbaldia procumbens* have previously been published in Allen *et al.* 2015 and Alsos *et al.* 2015, respectively, but then as part of other research questions.

**Table 2.** Sampling information and results for AFLP analyses, ordered from highest to lowest *D*-value for each species.

Taxon	Pop ID	Country^1^	Region	Locality	Latitude (N)	Longitude (E)	Year	Collector(s)^2^	*n*	*D*	% polym.	DW	Min–max	Private
*Botrychium lunaria*														
	Bl06	AT	Salzburg	Lungau	47.159191	13.380276	2009	AT	10	0.144	35.2	0.561	8–9	0
	Bl12	IT	Abruzzo	Roccaraso, Monte Pratello	41.799996	13.983328	2009	PK	10	0.142	40.8	0.653	10	1
	Bl13	CH	Bern	Wilderswil, Schynige Platte	46.652501	7.9138859	2009	PK	10	0.132	33.8	0.636	8–9	2
	Bl05	NO	Troms	Henrikheia, Tønsvikdalen	69.70651	19.242045	2009	TA	9	0.123	22.5	0.659	4–5	3
	Bl08	NO	Hedmark	Folldal gruver	62.141457	9.9923884	2010	RE, SB	10	0.122	23.9	0.388	6–8	0
	Bl14	NO	Troms	Kåfjord	69.385958	21.050901	2009	RE	5	0.124	21.1	0.446	5	0
	Bl07	IT	Piemonte	Rifugio Mongioie	44.16222	7.7827733	2009	AT	10	0.117	28.2	0.577	8–9	1
	Bl04	IS	Suðurland	Geysir	64.313761	−20.30295	2009	IEBS, SB	2	0.109	8.5	0.709	2	0
	Bl09	NO	Hordaland	Finse	60.601938	7.5038925	2010	IEBS, SB	10	0.089	23.9	0.428	8–9	1
	Bl11	FO (DK)	Eysturoy	Strendur	62.096476	−6.770503	2010	IEBS, JD	10	0.068	21.1	0.491	3–5	1
	Bl15	IS	Austurland	Skaftafell	64.015988	−16.972	2010	ÓBM	3	0.061	7.0	0.463	3	0
	Bl02	GL (DK)	Sermersooq	Tasiilaq	65.611727	−37.62076	2009	IEBS, SB	7	0.054	11.3	0.313	4–5	0
	Bl10	IS	Suðurnes	North of Kleifarvatn	63.869997	−22.55834	2010	RE	10	0.027	8.5	0.246	1–2	0
	Bl03	IS	Suðurland	Laugarvatn	64.213434	−20.77142	2009	IEBS, SB	2	0.018	1.4	0.185	1	0
	Bl01	**S (NO)**	Haakon VII Land	Bockfjorden	79.388374	13.439305	2009	IEBS, IGA, RE, SB	3	0.000	0.0	0.555	1	0
									μ	*0.089*	*19.1*	*0.487*		
									σ	*0.048*	*12.1*	*0.151*		
*Botrychium boreale*														
	Bb02	NO	Hedmark	Folldal			2010	IEBS, RE	5					
*Carex capillaris*														
*ssp.fuscidula*	Cc08	NO	Troms	Nordreisa II	69.507657	21.30714	2009	RE	4	0.199	36.1	1.308	4	0
*ssp.fuscidula*	Cc07	NO	Troms	Nordreisa I	69.606346	22.154588	2009	RE	4	0.157	27.7	1.012	4	0
*ssp.fuscidula*	Cc06	NO	Troms	Tromsø	69.525222	19.168228	2009	RE	3	0.120	18.1	1.015	3	0
*ssp.fuscidula*	Cc05	IS	Suðurnes	Grindavik	63.83144	−22.45722	2010	RE	7	0.119	26.5	0.978	7	0
*ssp.fuscidula*	Cc02	IS	Suðurland	Laugarvatn	64.211609	−20.77471	2009	IEBS, SB	10	0.049	16.9	0.924	6–8	1
*ssp.fuscidula*	Cc01	**S (NO)**	Haakon VII Land	Bockfjorden	79.385909	13.440977	2009	IGA, IEBS, RE, SB	10	0.044	13.3	1.022	8–10	1
*ssp. capillaris*	Cc03	IT	Valle d'Aosta	Walliser Alpen	45.941387	7.6550009		AT	8	0.139	34.9	0.978	7	1
*ssp. capillaris*	Cc10	CH	Valais	Zermatt	45.983891	7.7922214	2009	PK	7	0.109	28.9	1.082	7	1
*ssp. capillaris*	Cc09	NO	Hedmark	Folldal	62.33944	10.207651	2010	IEBS, RE, SB	9	0.104	24.1	1.333	9	3
Uncertain	Cc11	GL (DK)	A. P. Olsen Land	Zackenberg	73.999998	−22.00002	2010	OG	6	0.142	33.7	2.499	6	6
									μ	*0.118*	*26.0*	*1.215*		
									σ	*0.047*	*7.5*	*0.448*		
*Carex krausei*														
	Ck01	S (NO)	James I Land	Kapp Smith	78.66421	15.113933	–	IGA	8	–	–			
*Comastoma tenellum*^3^														
	Ct06	CH	Valais	Zermatt	45.983329	7.7833381	2009	PK	10	0.103	27.4	1.042	9	3
	Ct09	AT	Styria	Sölkpass	47.269996	14.080002	→	Schönswetter *et al*. (2004)	5	0.088	16.7	1.038	5	1
	Ct10	RU	Polar Ural	Slantzevaga mountain	66.905802	65.728889	2004	AT, IGA	10	0.042	10.7	1.035	4–9	2
	Ct07	CH/IT	Splügenpass		46.499998	9.3300063	→	Schönswetter *et al*. (2004)	5	0.038	7.1	1.035	4–5	3
	Ct02	NO	Troms	Tromsø	69.783802	19.435459	2009	TA	9	0.036	15.5	1.040	5–7	0
	Ct04	**S (NO)**	Ny-Fries Land	Ringhorndalen	79.335782	16.128264	2010	AKB, IGA	10	0.028	8.3	1.036	5–7	0^4^
	Ct01	**S (NO)**	Haakon VII Land	Ossian Sarsfjellet	78.928385	12.448045	2009	IGA, IEBS, RE, SB	7	0.024	7.1	1.036	2–3	0^4^
	Ct11	US	Alaska	Seward Peninsula	65.259997	−166.35	→	Schönswetter *et al*. (2004)	5	0.019	4.8	1.038	2–4	5
	Ct05	NO	Hedmark	Folldal	62.192569	9.7780116	2010	AKB, IEBS, RE, SB	10	0.018	6.0	1.036	2–4	0
	Ct03	**S (NO)**	Ny-Fries Land	Flatøyrdalen	79.289322	16.054733	2010	AKB, IGA, PBE, RE	8	0.011	2.4	1.032	1–4	0^4^
	Ct08	FR/IT	Col du Petit St. Bernard		45.669984	6.8699964	→	Schönswetter *et al*. (2004)	2	–	–			
									μ	*0.041*	*10.6*	*1.037*		
									σ	*0.031*	*7.0*	*0.003*		
*Gentianella campestris*														
	Gc01	NO	Troms	Tromsø	69.719201	19.102329	2009	TA	5	–	–	–	–	
*Kobresia simpliciuscula*														
ssp. *subholarctica*	Ks02	**S (NO)**	Haakon VII Land	Ossian Sarsfjellet	78.928538	12.459776	2009	IEBS, IGA, ÓBM, RE, SB	10	0.016	3.0	0.375	2–3	1
ssp. *subholarctica*	Ks04	**S (NO)**	Ny-Fries Land	Flatøyrdalen	79.285726	16.028505	2010	AKB, IGA, PBE, RE	9	0.009	1.5	0.365	1–2	1
ssp. *subholarctica*	Ks01	**S (NO)**	Bünsow land	Gipsvika	78.453216	16.535147	2009	IEBS, IGA, SB	9	**0.000**	**0.0**	0.277	1	0
ssp. *subholarctica*	Ks03	**S (NO)**	Haakon VII Land	Blomstrand	78.973786	12.184115	2009	IEBS, IGA, ÓBM, RE, SB	10	**0.000**	**0.0**	0.277	1	0
ssp. *simpliciuscula*	Ks05	NO	Sør-Trøndelag	Røros	62.60159	11.454584	2009	RE	5	0.120	9.1	1.228	3	2
ssp. *simpliciuscula*	Ks06	NO	Hedmark	Folldal	62.317515	9.8531303	2010	AKB, IEBS, RE, SB	8	0.020	3.0	0.819	2–3	0
									μ	*0.028*	*2.8*	*0.557*		
									σ	*0.046*	*3.1*	*0.353*		
*Kobresia myosuroides*														
	Km02	IS	Suðurland	Geysir			2009	IEBS, SB	5					
*Ranunculus wilanderi*														
	Rw01^5^	**S (NO)**	Dickson Land	Kapp Thordsen 2009	78.459342	15.545322	2009	IEBS, IGA, ÓBM, RE, SB	11	0.001	0.9	–	1–2	–
	Rw02^5^	**S (NO)**	Dickson Land	Kapp Thordsen 2008	78.459055	15.52601	2008	IGA	8	–				
*Ranunculus auricomus*														
	Ra01	NO	Hedmark	Folldal			2010	AKB, IEBS, RE, SB	5					
*Sibbaldia procumbens*^3^														
	Sp16	GL (DK)	Qaasuitsup	Blomsterdalen, Qeqertarsuaq (Disko island)	69.889429	−53.5038796	2006	KBW	4	0.239	25.2	1.024	4	2
	Sp05	GL (DK)	Sermersooq	Kulusuk	65.575276	−37.18333	2009	IEBS, SB	4	0.108	11.7	0.300	4	0
	Sp17	RU	Khanty-Mansia	The Ural Mountains	60.7119262	65.3411036	2004	IGA, AT	5	0.100	12.6	1.406	5	4
	Sp12	IS	Vestfirðir	Brekkudalur, Önundarfjördur	66.0499747	−23.5833233	2010	RE	10	0.095	14.4	0.391	10	0
	Sp04	GL (DK)	Sermersooq	Tasiilaq	65.619883	−37.66083	2009	IEBS, SB	10	0.090	15.3	0.290	6–8	0
	Sp19	US	Alaska	Unalaska	53.8896998	−166.384296	2007	BK	5	0.087	10.8	0.753	5	2
	Sp11	NO	Hordaland	Finse	60.601938	7.5038925	2010	IEBS, SB	10	0.068	11.7	0.314	8–9	0
	Sp20	CA	Yukon	North Yukon	67.8999928	−136.56667	2006	BB	5	0.065	8.1	0.383	5	0
	Sp10	NO	Hedmark	Folldal	62.311994	9.8472831	2010	AKB, IEBS, RE, SB	10	0.063	9.0	0.296	6–8	0
	Sp13	FO (DK)	Eysturoy	Strendur	62.1242772	−6.78213456	2010	IEBS, JD	8	0.052	7.2	0.307	8	0
	Sp09	IT	Piemonte	Colle dell’Agnello	44.678596	6.9886156	2009	AT	10	0.043	10.8	0.429	4–6	1
	Sp21	J (NO)	South-West Jan Mayen	Unknown	70.9833308	−8.53333865	2011	GA	3	0.032	2.7	0.283	2	0
	Sp14	CH	Valais	Aletschgletscher	46.4061104	8.07222789	2009	PK	10	0.027	5.4	0.604	5–7	2
	Sp07	AT	Salzburg	Lungau, Oberes Murtal, Radstädter Tauern	47.157294	13.372774	2009	AT, KM	10	0.023	4.5	0.367	3-4	
	Sp08	IT	Valle d’Aosta	Breuil-Cervínia, Plan Maison	45.941387	7.6550009	2009	AT	10	0.018	2.7	0.284	3–4	0
	Sp06	IS	Austurland	Skaftafell	64.016111	−16.97194	2010	ÓBM	11	0.015	4.5	0.314	2–4	0
	Sp01Sp02Sp03	**S (NO)**	Haakon VII Land	Bockfjorden	79.390688	13.435951	2009	IEBS, IGA, RE, SB	25	**0.000**	**0.0**	0.300	1	0
	Sp15	CA	Nunavut	Kitikmeot region	67.0038353	−110.008489	2006	BA	1	–	–	–	1	0
									μ	*0.066*	*9.2*	*0.473*		
									σ	*0.055*	*5.9*	*0.304*		
*Sibbaldia cuneata*														
	Sc01						2009	RE	2					
*Tofieldia pusilla*														
	Tp07	GL (DK)	Sermersooq	Tasiilaq	65.609851	−37.61969	2009	IEBS, SB	10	0.214	53.1	0.290	9–10	1
	Tp13	DE	Berchtesgadener	Watzmann	47.5683175	12.9316674	2009	AT	5	0.213	40.6	0.197	5	0
	Tp10	NO	Troms	Tromsø	69.786215	19.448587	2009	TA	9	0.207	53.1	0.183	9	0
	Tp05	**S (NO)**	Haakon VII Land	Bockfjorden	79.389574	13.439082	2009	IGA, IEBS, RE, SB	6	0.173	40.6	0.196	6	0
	Tp04	**S (NO)**	Dickson Land	Kapp Nathorst	78.772072	15.45458	2009	EM, IEBS, SB	10	0.156	40.6	0.196	10	0
	Tp15	NO	Hedmark	Folldal	62.140768	9.9895844	2010	IEBS	9	0.151	31.3	0.184	7–8	0
	Tp16	**S (NO)**	Ny-Fries Land	Flatøyrdalen	79.287063	16.046994	2010	AKB, IGA	8	0.144	37.5	0.188	6–8	0
	Tp01	**S (NO)**	Dickson Land	Blomesletta	78.619192	14.841571	2009	IGA, IEBS, SB	9	0.139	37.5	0.191	7–9	0
	Tp09	IS	Suðurland	Geysir	64.314253	−20.30637	2009	IEBS, SB	10	0.139	34.4	0.231	8–9	0
	Tp03	**S (NO)**	Dickson Land	Kapp Wijk	78.5981	15.330918	–	IGA, KBW	10	0.133	34.4	0.203	6–9	0
	Tp12	NO	Troms	Nordreisa	69.706802	21.209988	2009	RE	5	0.119	28.1	0.220	3–5	0
	Tp19	IS	Vestfirðir	Önundarfjördur	65.9823825	−23.3805798	2010	RE	5	0.106	21.9	0.184	4–5	0
	Tp18	**S (NO)**	Haakon VII Land	Ossian Sarsfjellet	78.937687	12.438682	–	IGA	5	0.094	21.9	0.214	5	0
	Tp02	**S (NO)**	Haakon VII Land	Blomstrand	78.974056	12.18039	2009	IGA, IEBS, SB	15	0.079	25.0	0.220	5–8	0
	Tp14	NO	Hordaland	Finse	60.606467	7.5489479	2010	IEBS	9	0.078	21.9	0.229	4–6	0
	Tp17	**S (NO)**	Ny-Fries Land	Ringhorndalen	79.333688	16.127416	2010	AKB, IGA	9	0.069	21.9	0.207	3–6	0
	Tp08	IS	Suðurland	Laugarvatn	64.211186	−20.77418	2009	IEBS, SB	9	0.052	12.5	0.259	2–6	0
	Tp11	AT	Salzburg	Weisseck	47.160879	13.3774409	2009	AT	2	–	–	–	–	–
									μ	*0.133*	*32.7*	*0.211*		
									σ	*0.050*	*10.9*	*0.028*		
*Tofieldia calyculata*														
	Tca01	AT	Salzburg	Lungau	47.174363	13.374747	–	AT, IGA	4					
*Tofieldia coccinea*														
	Tco01	CA	Yukon	Yukon/Northwest Territories	67.039121	−136.21555	–	LG	8					

Headings: population identity [Pop ID]; number of individuals sampled for AFLP analyses [*n*]; average proportion of pairwise differences [*D*]; proportion of polymorphic markers [% polym.]; frequency down weighed marker value [DW]; minimum to maximum number of AFLP multilocus phenotypes [min–max]; number of private AFLP markers [Private]. Mean [μ] and standard deviation [σ] for [D], [% polym.] and [DW] given below each species. Populations from Svalbard are indicated in bold. Outgroups for neighbuor-joining analyses marked in grey.

^1^Country: AT, Austria; CA, Canada; CH, Switzerland; DE, Germany; DK, Denmark; FO, Faroe Islands; FR, France; GL, Greenland; IS, Iceland; IT, Italy; J, Jan Mayen; NO, Norway; RU, Russia; S, Svalbard; US, United States of America.

^2^Collectors: AKB, Anne Krag Brysting; AT, Andreas Tribsch; EM, Eike Müller; BA, Brian Apland; BB, Bruce Bennett; BK, Brad Krieckhaus; GA, Geir Arnesen; IGA; Inger Greve Alsos; IEBS, Idunn Elisabeth Borgen Skjetne; JD, Jan Djurhuus; KBW, Kristine Bakke Westergaard; KM, Karin Moosbrugger; LA, Liudmila Aleksandrovha Sergienko; LG, Lovisa Gustafsson; ML, Maarten J. J. E. Loonen; OG, Olivier Gilg; ÓBM, Ólöf Birna; Magnúsdóttir; PBE, Pernille Bronken Eidesen; PK, Patrick Kuss; RE, Reidar Elven; SB, Siri Birkeland; TA, Torbjørn Alm; UL, Unni Lundgren.

^3^*Comastoma tenellum* plant material from the Alps and Alaska is from Schönswetter *et al*. (2004) and plant material and AFLP data for *S. procumbens* has previously also been published in Allen *et al*. (2015) and Alsos *et al*. (2015a), respectively, but then as part of other research questions.

^4^Two private markers were found in Svalbard as a whole when grouping Ct01, Ct03 and Ct04.

^5^Rw01 and Rw02 represent one population and were therefore pooled in the analyses.

### DNA isolation

Approximately 20 mg of silica dried leaves were placed in 2 ml tubes with two tungsten carbon beads and crushed at 20 Hz for 2–8 min on a mixer mill (MM03, Retsch GmbH & Co, Haan, Germany). To obtain optimal purity and concentration of DNA, two to three different extraction protocols were tested on a few individuals of each species, and the best protocol was used further. DNA from the individuals of *Botrychium lunaria*, *Carex capillaris*, *Kobresia simpliciuscula* and *Sibbaldia procumbens* was isolated using the acidic DNA isolation protocol by Ziegenhagen *et al.* (1993) with the following modifications: The silica dried leaves were crushed to powder as explained above, without the use of liquid nitrogen. The samples were quickly spun down before a preheated (65 °C) extraction buffer was added. The first centrifugation step was increased to 15 min at 13 000 rpm, the second centrifugation step was increased to 20 min at 13 000 rpm and the last centrifugation step was increased to 15 min at 13 000 rpm. In addition, an extra purification step was added after the last centrifugation: 1 ml ice-cold 70% ethanol was added to each sample, centrifuged for 2 min at 13 000 rpm, and then removed. This step was repeated before the samples were left over night to dry. The final DNA pellet was dissolved in 100 µl TE-buffer and 1 µl RNAse was added before the incubation at 37 °C. DNA from individuals of *Comastoma tenellum* and *Ranunculus wilanderi* was isolated using the Qiagen DNeasy™ Plant Mini Kit (Qiagen, Hilden, Germany), following the manufacturer’s protocol. DNA from *Tofieldia pusilla* individuals was isolated using the E.Z.N.A.™ SP Plant DNA Mini Kit, following the protocol for dry specimens (Omega Bio-Tek, Norcross, USA). The protocol was modified by adding a freezing step (at −80 °C for 10 min) prior to cell lysis. To increase the final DNA concentration of *C. tenellum* and *T. pusilla* samples, the amount of AE buffer was reduced to 30–50 µl, the first eluate (i.e. DNA dissolved in AE buffer) was re-eluted in a second elution step, and incubation was done at 65 °C. The DNA concentration of the samples was measured using a spectrophotometer (NanoDrop™ 1000, Thermo Fisher Scientific, Wilmington, USA). Samples isolated with the Ziegenhagen protocol were diluted so that the final DNA concentrations were approximately the same within each species (60 ng/µl). The Qiagen DNeasy Plant Mini Kit and the E.Z.N.A. Plant DNA Mini Kit gave concentrations of approximately 20 ng/µl, which were kept undiluted.

### AFLP analysis

Amplified Fragment Length Polymorphism (AFLP) was used to generate dominant molecular markers from the sampled individuals (Vos *et al.* 1995). The AFLP procedure was modified slightly from Jørgensen *et al.* (2006): 2 µl DNA isolate was used in the restriction-ligation step, and the amount of AmpliTaq polymerase (Applied Biosystems/Life Technologies, Carlsbad, CA, USA) used in the pre-selective amplification of fragments was increased to 0.075 µl. PCR conditions during the elongation step were modified to 2 and 1 min at 72 °C for the pre-selective and selective amplification of fragments, respectively. All reactions were carried out on an Eppendorf Thermal Cycler (Mastercycler® ep gradient S, Hamburg, Germany). A series of primer tests were performed prior to the final selective amplification step on a selection of samples from different geographic regions [**see Supporting Information—Table S3**]. Finally, 3–4 primer pairs were chosen for each species [**see Supporting Information—Table S3**]. The 6-FAM EcoRI-primer and all non-labelled primers and adaptors were ordered from MWG (Ebersberg, Germany) or IDT (Leuven, Belgium), while the other fluorescent-labelled primers were ordered from Applied Biosystems/Life Technologies. A set of negatives, replicates and duplicates was included in all final AFLP runs to check for contamination and replicability (Bonin *et al.* 2004). The fluorescently labelled AFLP fragments were detected on an ABI3730 DNA Analyser (Applied Biosystems/Life Technologies). For each sample, 2 µl from a mix of co-loaded selective products (3 µl FAM, 3 µl NED, 3 µl PET and 2 µl VIC) were mixed with 0.3 µl GeneScan™ 500 (-250) LIZ size standard and 11.7 µl Hi-Di™ formamide (both from Applied Biosystems/Life Technologies). Selective products of *Sibbaldia procumbens* were run with only 8.85 µl HiDi formamide and 0.15 µl LIZ size standard. The plate was vortexed, spun down and denatured at 95 °C for 5 min. After denaturation, the plate was immediately put on ice for a few minutes and then run on the ABI Analyzer.

AFLP profiles were visualized using GeneMapper ver. 4.0 (Applied Biosystems). Unambiguously scorable fragments (peaks) in the size range of 50–500 bp were scored as absence/presence, following the approach of Whitlock *et al.* (2008), and their R-based interactive scripting program AFLPscore ver. 1.4., using the filtering option for locus selection and relative threshold for phenotype calling. Error rate estimation was calculated as the average percentage of differences between replicate pairs (i.e. mismatch error rate; Bonin *et al.* 2004). For each primer combination, the thresholds for locus selection and phenotype calling that resulted in the highest number of highly reproducible markers were chosen. Fragments with a frequency lower than the error rate were re-checked and removed if no clear peak was present. Fragments missing in only a few individuals were also re-checked and corrected if scored incorrectly.

### Statistical analyses of AFLP data

The percentage of polymorphic AFLP markers was calculated both at species level [**see Supporting Information—Table S3**] and at population level. Monomorphic markers at species level were excluded from further analyses. Within-population genetic diversity was estimated as the average proportion of pairwise differences between individuals, D (Nei 1973; Kosman 2003) and the percentage of polymorphic markers. The minimum and maximum number of AFLP multilocus phenotypes was calculated for each population. The minimum number of AFLP multilocus phenotypes included only multilocus phenotypes which were identical across all markers, whereas the maximum number of multilocus phenotypes allowed for a number of pairwise differences equal to the error rate. To address the genetic distinctiveness of the Svalbard populations, ‘frequency down weighted marker values’ (DW) were calculated according to Schönswetter and Tribsch (2005) (except for populations with less than two sampled individuals). Private AFLP markers (i.e. markers unique to the Svalbard populations) were also recorded. All calculations listed above, as well as most data format conversions, were performed using the AFLPdat R-script ver. 2010 (Ehrich 2006) in R ver. 3.2.1 (R Core Team 2015).

Genetic groups were delineated for each species (except *Ranunculus wilanderi*) using STRUCTURE ver. 2.3.3 (Pritchard *et al.* 2000), run through the Bioportal (now the Lifeportal) of the University of Oslo. We applied the no-admixture model on the AFLP data, which was treated as diploid multi-locus genotypes, using the recessive allele model for dominant markers (Falush *et al.* 2007). The number of possible groups, K, was set to range from one to the total number of sampling localities for each species. Ten independent runs were carried out for each number of K. A burn-in period of 10^5^ iterations was followed by 10^6^ iterations. The results of the independent runs were summarized using the R-script STRUCTURE-sum ver. 2011 (Ehrich *et al.* 2007) and the most appropriate number of genetic groups, K, was determined according to recommendations in Evanno *et al.* (2005; i.e. as the K with the highest value of delta K), but posterior probabilities (Pritchard *et al.* 2000) and similarity coefficient estimates (Nordborg *et al.* 2005) were also considered. To reveal hierarchical genetic structure in the data, separate STRUCTURE analyses were run on the group(s) to which the Svalbard individuals were grouped by the first STRUCTURE analysis for species with moderate to strong geographic structure. Finally, supplementary principal coordinates analyses (PCO) (Davis 1986) and neighbour-joining analyses (Saitou and Nei 1987) were performed to evaluate the results obtained by the STRUCTURE analyses. PCO and neighbour-joining analyses were performed in PAST ver. 2.13 (Hammer *et al.* 2001) using the Dice similarity coefficient (Dice 1945). Most results from the PCO and neighbour-joining analyses are not presented, as they were largely congruent with the STRUCTURE results. However, the neighbour-joining and PCO analyses gave support for a separate Greenlandic group in *Carex capillaris* (for PCO plot, [**see Supporting Information—Figure S4]**), contradicting the results from STRUCTURE. Due to its uncertain affiliation, the Greenlandic population was omitted from further analyses (i.e. the hierarchical STRUCTURE analysis, AMOVA analyses and the assignment tests).

To determine the partitioning of genetic variation among populations and among genetic groups revealed by the STRUCTURE analyses, AMOVAs (analyses of molecular variance) were run in Arlequin ver. 3.5 (Excoffier *et al.* 2005). A fixation index, the F_ST_ analogue for dominant markers (Φ_ST_; Excoffier *et al.* 1992), was calculated based on the number of pairwise differences between individuals.

The source area(s) of the Svalbard populations (except for *Ranunculus wilanderi* and *Kobresia simpliciuscula*) was inferred by performing multi-locus assignment tests in AFLPOP ver. 1.1 (Duchesne and Bernatchez 2002). Geographically consistent genetic groups or subgroups (i.e. obtained by the STRUCTURE analyses) were considered as potential source areas. If no geographic genetic structure was revealed by the STRUCTURE and additional PCO and neighbour-joining analyses, geographic regions were considered as potential source areas. We used a log likelihood difference of one as a threshold for allocation. With this threshold, the likelihood for an AFLP phenotype to be found in its most likely source region had to be 10 times higher, or more, than for the second most likely source region.

As in Alsos *et al.* (2007), we examined the genetic founder and bottleneck effects in relation to adaptation to the current climatic conditions in Svalbard. We used six different measures to quantify the genetic founder/bottleneck effects (Alsos *et al.* 2007; **[see Supporting Information—Table S5]**). To quantify the adaptation to the current climatic conditions in Svalbard, we used two measures of temperature requirement and rated their rarity (Alsos *et al.* 2007; [**see Supporting Information—Table S5]**). The measures of genetic founder/bottleneck effects and climatic adaptation were summarised in two separate principal component analyses (PCA), using R ver. 3.2.1 (R Core Team 2015). The first principal components from the two analyses were then plotted against each other, showing the genetic founder/bottleneck effects for the species in relation to their adaptation to the current climatic conditions in Svalbard. Finally, a simple linear regression was performed to find the correlation coefficient between the two variables (i.e. climatic adaptation and founder/bottleneck effects). *Ranunculus wilanderi* was omitted from the analysis due to limited AFLP data. In addition to the study species, we included 12 species with already published AFLP data from Svalbard (Alsos *et al.* 2007; Westergaard *et al.* 2011; Gussarova *et al.* 2012; [**see Supporting Information—Table S5**]).

## Results

### Number of populations, population sizes and red list categories

The number of populations found in Svalbard (Table 1) ranged from one (*Botrychium lunaria*, *Carex capillaris* ssp. *fuscidula*, *Ranunculus wilanderi* and *Sibbaldia procumbens*) to ten (*Tofieldia pusilla*); all populations were situated within the warmest bioclimatic subzone in Svalbard (the Middle Arctic Tundra Zone). Two populations of *T. pusilla* and one of *Comastoma tenellum* were previously unknown. The population sizes ranged from less than five individuals (*T. pusilla*, Ossian Sarsfjellet) to more than 2000 (*C. capillaris* ssp. *fuscidula*, Bockfjorden). These new population size data led to a downgrading of *C. capillaris* ssp. *fuscidula*, *T. pusilla*, *S. procumbens*, *C. tenellum* and *R. wilanderi* in the 2010 Red List [**see Supporting Information—Table S1**]. However, *T. pusilla* was upgraded from ‘Least Concern’ to ‘Near Threatened’ in the 2015 Red List due to a higher weighting of fragmentation of its range. The same year, an adjustment to the IUCN criteria also led to a further downgrading of *C. tenellum* (now ‘Vulnerable’) and *C. capillaris* (now ‘Near Threatened’). At present, five of the seven study species are considered threatened in Svalbard [**see Supporting Information—Table S1**], mostly due to restricted extent of occurrence (criterion B1), limited area of occupancy (criterion B2) and/or a low number of reproducing individuals (criterion D1) (Henriksen and Hilmo 2015).

### Genetic results

**Figure 2:**
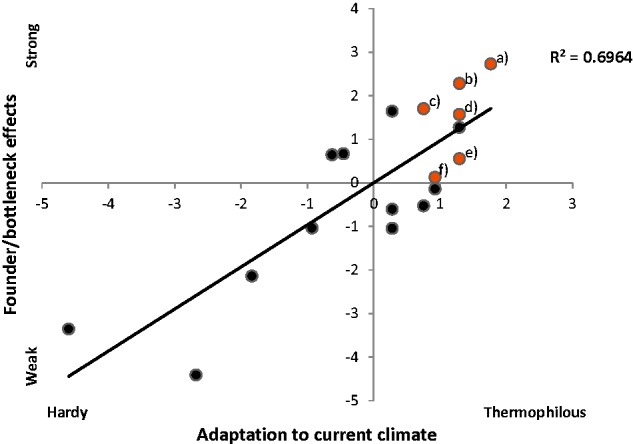
Index of genetic founder/bottleneck effects plotted against index of adaptation to the current climatic conditions in Svalbard for six of the study species analysed in comparison to 12 additional species. The axes are the first principal components from two separate principal component analyses, summarizing three measures of climatic adaptations and six measures of genetic founder/bottleneck effects based on AFLP data [**see Supporting Information—Table S5**]. The axes are explaining 90.3% and 60.3% of the total variation, respectively. The study species are indicated with letters: (a) *Botrychium lunaria*; (b) *Sibbaldia procumbens*; (c) *Kobresia simpliciuscula* ssp. *subholarctica*; (d) *Carex capillaris* ssp. *fuscidula*; (e) *Comastoma tenellum* and (f) *Tofieldia pusilla*.

The levels of genetic variation within the Svalbard populations were low for most species, with only one AFLP multilocus phenotype identified in *Botrychium lunaria*, *Sibbaldia procumbens* and probably also in *Ranunculus wilanderi* (Table 2). Moreover, there was a positive correlation between genetic founder/bottleneck effects and thermophily (*R*^2 ^=^ ^0.6964, *n* = 18, Fig. 2). The strongest founder/bottleneck effects were found in *B. lunaria*, which is also the most thermophilous species [**see Supporting Information—Table S5**]. Strong founder/bottleneck effects and high levels of thermophily were also found in *S. procumbens, Carex capillaris* ssp. *fuscidula* and *Kobresia simpliciuscula* ssp. *subholarctica*. Intermediate levels of founder/bottleneck effects were found in *Tofieldia pusilla* and *Comastoma tenellum*.

*Tofieldia pusilla* and *Botrychium lunaria* had a considerably higher proportion of within population genetic variation than among population genetic variation according to the AMOVA (Table 3). Furthermore, the STRUCTURE analyses delineated four genetic groups in *T. pusilla* (*K* = 4) and three genetic groups in *B. lunaria* (*K* = 3). However, *T. pusilla* (Fig. 1a) had the weakest geographic pattern with nearly all populations being admixed, while admixture occurred only in half of the *B. lunaria* populations (Fig. 1b). Neither species had unique STRUCTURE groups nor private markers in Svalbard. The assignment tests could not target source area(s) for the Svalbard population of *B. lunaria*. For *T. pusilla*, the assignment test allocated the Svalbard populations to a large unspecified European group including all sampled populations except Greenland [**see Supporting Information—Table S6]**.

**Table 3.** Analyses of molecular variance (AMOVAs) of AFLP multilocus phenotypes in six of the seven study species.

Species	Source of variation	d.f.	% of total variation	Fixation index (*Φ*_ST_)*
*Tofieldia pusilla*	Among all populations	17	35.4	
	Within all populations	127	64.6	0.35
*Botrychium lunaria*	Among all populations	14	36.1	
	Within all populations	97	63.9	0.36
*Carex capillaris*	Among all populations	9	65.9	
	Within all populations	58	34.1	0.66
	Among two main genetic groups (largely corresponding to ssp. *capillaris* and ssp. *fuscidula*)	1	28.0	
	Among populations within two main genetic groups (largely corresponding to ssp. *capillaris* and ssp. *fuscidula*)	7	38.6	
	Within populations	53	33.5	0.67
*Sibbaldia procumbens*	Among all populations	17	78.3	
	Within all populations	133	21.7	0.78
	Among two main genetic groups	1	69.9	
	Among populations within two main genetic groups	16	15.7	
	Within populations	133	14.4	0.86
*Comastoma tenellum*	Among all populations	10	86.2	
	Within all populations	70	13.8	0.86
	Among three main genetic groups	2	57.0	
	Among populations within three main genetic groups	8	31.4	
	Within populations	70	11.5	0.88
*Kobresia simpliciuscula*	Among all populations	5	94.6	
	Within all populations	45	5.4	0.95
	Among two main genetic groups (corresponding to ssp. *subholarctica* and ssp. *simpliciuscula*)	1	94.5	
	Among populations within two main genetic groups (corresponding to ssp. *subholarctica* and ssp. *simpliciuscula*)	4	2.9	
	Within populations	45	2.6	0.97

Headings: components of variance [Source of variation]; degrees of freedom [d.f.]; percentage of total variation [% of total variation]; fixation index for binary data [Fixation index (*Φ*_ST_)]. (main genetic groups were first of all inferred from STRUCTURE analyses (see Fig. 2), but PCO and neighbour-joining analyses were also considered).

* All *P* < 0.0001.

*Carex capillaris* and *Sibbaldia procumbens* both had a considerably higher proportion of among population genetic variation than within population genetic variation according to the AMOVA (Table 3). When taking the two STRUCTURE groups found in *S. procumbens* (Fig. 1c) into account, as much as 69.9 % of the total detected genetic variation was attributed to variation among these (Table 3). In the hierarchical STRUCTURE analysis of the Eurasian group (data not shown), the Svalbard population of *S. procumbens* formed a group together with the Russian population and a population from Folldal, mainland Norway (hereafter called Northwest Europe). Two main STRUCTURE groups (*K* = 2) were also delineated in *C. capillaris* (Fig. 1d), largely corresponding to the two assumed subspecies *C. capillaris* ssp. *fuscidula* and *C. capillaris* ssp. *capillaris*. In the hierarchical STRUCTURE analysis of the ssp. *fuscidula* group (data not shown), the Svalbard population was separated as its own group. The assignment test confirmed Northwest Europe and Northern Norway as the source areas for the Svalbard individuals of *S. procumbens* and *C. capillaris* ssp. *fuscidula*, respectively. However, source area was only confirmed in half of the *C. capillaris* ssp. *fuscidula* individuals [**see Supporting Information—Table S6**]. In both *C. capillaris* ssp. *fuscidula* and *S. procumbens*, the Svalbard population scored below average on the rarity index (DW = 1.022 and 0.300, respectively, Table 2), but one private Svalbard marker was found in *C. capillaris* ssp. *fuscidula* (Table 2).

*Comastoma tenellum* had high among population variation (Table 3) and the STRUCTURE analysis revealed three geographically consistent genetic groups: (1) Svalbard and Russia, (2) Alaska, Norway and one population from the Alps and (3) the remaining populations from the Alps (Fig. 1e). The assignment tests indicated Russia as source area for all Svalbard individuals [**see Supporting Information—Table S6**], but Svalbard constituted a separate group in the additional hierarchical STRUCTURE analysis for the Svalbard-Russia group (data not shown). Furthermore, two private Svalbard markers were also found (Table 2).

Two STRUCTURE groups were delineated for *K. simpliciuscula* (Fig. 1f). These two groups corresponded to ssp. *simpliciuscula* and ssp. *subholarctica*, and nearly all detected genetic variation in the data set was attributed to variation between these two subspecies (Table 3). Two of the *K. simpliciuscula* populations in Svalbard (Ossian Sarsfjellet and Flatøyrdalen) possessed one possible private marker each (Table 2). Finally, STRUCTURE and AMOVA analyses were not performed for the endemic and genetically depauperate microspecies *R. wilanderi* (Fig. 1g).

## Discussion

As expected from Alsos *et al.* (2007), we found that genetic founder/bottleneck effects are correlated with adaptation to the climatic conditions in Svalbard. Furthermore, we found that most of our study species, which are characterized by high levels of thermophily, have experienced strong genetic founder/bottleneck effects. Climatic limitations seem also to be reflected in the number, sizes and localization of the examined Svalbard populations.

### Causes of low levels of genetic variation

Alsos *et al.* (2007) interpreted the stronger genetic founder/bottleneck effect in thermophilous plants in Svalbard as a result of restricted establishment, survival and local reproduction rather than dispersal per se. Temperature has probably been less of a limiting factor for thermophilous species arriving in the early Holocene warm period, as previously inferred for e.g. *Betula nana, Campanula rotundifolia*, *Vaccinium uliginosum* (Alsos *et al.* 2002), *Euphrasia wettsteinii* (Gussarova *et al.* 2012) and *Salix herbacea* (Alsos *et al.* 2009). The observed genetic patterns are therefore likely a product of subsequent bottleneck effects following climate cooling rather than an initial founder effect for this group of species. Most of our study species probably belong to the group of early Holocene arrivals, and some of them even have populations that are clearly differentiated from their source populations outside Svalbard. The Svalbard populations of *Carex capillaris* ssp. *fuscidula* and *Comastoma tenellum* were for instance identified as unique groups in the hierarchical STRUCTURE analyses and also harboured one and two private markers, respectively. Colonization during the warmer parts of the Holocene can also be inferred for *Kobresia simpliciuscula* ssp. *subholarctica* and *Tofieldia pusilla* as these two species have multiple populations with several AFLP multilocus phenotypes despite today’s unfavourable climate.

In contrast, the single populations of *Botrychium lunaria* and *Sibbaldia procumbens* consisted only of one AFLP multilocus phenotype and were not differentiated from populations in other geographic regions. *Botrychium lunaria* and *S. procumbens* also showed the strongest genetic founder/bottleneck effects of all species included. It is somewhat surprising to observe such a strong founder/bottleneck effect in *B. lunaria* as we expected levels of genetic variation to be extremely low throughout the distribution range due to intragametophytic self-fertilization (see e.g. Soltis *et al.* 1988; Hauk and Haufler 1999; Farrar 2006). Contrary to what we predicted, most *B. lunaria* populations actually contain many AFLP multilocus phenotypes and a higher proportion of within population genetic variation relative to among population genetic variation. This pattern has, however, also been found in several other *Botrychium* studies that are using non-coding markers (Camacho and Liston 2001; Williams 2012). As there is generally low genetic differentiation among *Botrychium* populations, the explanation is probably a combination of high dispersal potential and a mainly inbreeding mating system (Soltis *et al.* 1988; Stensvold 2008; Williams 2012). The strong genetic founder/bottleneck effects in *B. lunaria* and *S. procumbens* may be the result of recent founding events and the observed lack of genetic diversity might suggest that each of their populations in Svalbard was established by a single propagule.

Overall, our results strongly support that the genetic depletion of the thermophilous species in Svalbard is a result of restricted initial establishment and/or population decline following climate cooling (Alsos *et al.* 2002, 2015), as well as lack of sexual reproduction under the present climatic conditions (Engelskjøn *et al.* 2003).

### Threats to the Svalbard populations

Due to low levels of genetic diversity, the thermophilous plant species in Svalbard may be vulnerable to inbreeding depressions and also have reduced evolutionary potential. This will however depend on species traits and species history. The risk of inbreeding depression may for instance be low for *Botrychium lunaria* as this is a pteridophyte that reproduces by intragametophytic self-fertilization and is expected to have undergone purging of deleterious recessive alleles (Farrar 2006). Similarly, *Ranunculus wilanderi* is apomictic and will not experience any increase in homozygosity with decreasing population size (Richards 2003; Pellino *et al.* 2013). Furthermore, like many other pteridophytes, the subterranean, gametophytic phase of *B. lunaria* is also highly dependent on its mycorrhizal fungal partner (Farrar 2006; Winther and Friedman 2007). The gametophyte is therefore thought to have reduced direct interaction with the environment and evolutionary potential may not entirely depend on genetic variation in the sporophyte generation (Farrar 2006). However, based on the results presented here, most of the study species may still be prone to inbreeding depressions, further loss of genetic variation and also have reduced adaptability to future environmental change.

In addition to the abovementioned threats, demographic and/or environmental stochasticity may also be of serious concern for the thermophilous plant species in Svalbard. This regards especially the species with few and small populations. Presence of seed banks may function as a buffer against population fluctuations and extinctions, but are not reported from thermophilous species in Svalbard (Alsos *et al.* 2003; Cooper *et al.* 2004). The relative extinction risk associated with demographic and/or environmental stochasticity will also depend on the population growth rate (Lande 1993). Future climate change may stimulate population growth, but this will depend on a number of factors like e.g. current reproductive fitness and habitat preferences. Arctic wetland species like *Carex capillaris* ssp. *fuscidula*, *Kobresia simpliciuscula* ssp. *subholarctica*, *Ranunculus wilanderi* and *Tofieldia pusilla* are for instance expected to be negatively affected by changes in drainage conditions, evaporation rates and water supply (Young *et al.* 1997). Furthermore, competition is expected to increase with climate warming, and Arctic species with conservative nutrient-use strategies, slow growth and inflexible morphologies may become outcompeted by more responsive, faster growing, taller species immigrating from southern latitudes (Callaghan *et al.* 2005). Tracking of potential population size changes may give valuable insights into climate change responses and, following, future extinction risk.

### Svalbard management units and an evolutionarily significant microspecies

The low levels of genetic diversity and distinctiveness that we recorded for the Svalbard populations of our study species are also reflected in most Arctic species studied until now, and may partly relate to the recent glaciation of the region (Eidesen *et al.* 2013; Stewart *et al.* 2016). We argue that all Svalbard populations examined in this study should be viewed as separate management units for three reasons: First, most of our study species have probably been present in Svalbard since the early Holocene warm period and for *Carex capillaris* ssp. *fuscidula* and *Comastoma tenellum* the Svalbard populations are genetically clearly differentiated from their source populations outside Svalbard (see above). Second, all examined Svalbard populations are likely demographically independent as there seems to be little current gene flow between these populations and populations outside Svalbard. This is clearly demonstrated by the strong founder/bottleneck effects. Finally, conservation of edge populations may be important for maintaining evolutionary potential as e.g. stress tolerance alleles may be more common here than in more optimal habitats (Sherwin and Moritz 2000). Considering the Svalbard populations as separate management units is also in line with the regional red list which treats Svalbard as a separate management area (Henriksen and Hilmo 2015).

Although delineating Evolutionarily Significant Units (ESUs) is beyond the scope of this study due to the lack of adaptive markers, information on *Ranunculus wilanderi* clearly suggests that it constitutes such a unit. The species is considered an endemic for the archipelago, but is just one of numerous microspecies within the *Ranunculus auricomus* complex (Jonsell 2001). Members of this complex possess the ability to produce seeds asexually by agamospermy (Jonsell 2001; Pellino *et al.* 2013), and reproductive isolation can therefore occur rapidly. *Ranunculus wilanderi* is nevertheless the only member of the *R. auricomus* complex present in Svalbard, it differs morphologically from other members in the *R. auricomus* complex (personal observation), and only shares its unusual habitat preference (damp moss tundra) with one other member from the complex; the diploid, and probably sexually reproducing, *Ranunculus boecheri* from eastern Greenland (Elven *et al.* 2011). Based on this we argue that *R. wilanderi* can be considered a separate ESU, although the relationship to other *R. auricomus* microspecies should be further examined.

### Genetic relationships of importance for conservation

If it should become necessary to consider management strategies like translocations, information about genetic relationships will be especially important for species with historically isolated populations and little to moderate contemporary gene flow (Ottewell *et al.* 2016). In our case, this relates especially to *Carex capillaris* ssp. *fuscidula*, *Sibbaldia procumbens* and *Comastoma tenellum*. Although Svalbard is known to be predominantly colonized from Northern Russia and only occasionally from Northern Norway and Greenland (Alsos *et al.* 2007; Gussarova *et al.* 2012; Alsos *et al.* 2015), we were only able to confirm Russia as source area for *C. tenellum*. For *C. capillaris* ssp. *fuscidula* the Svalbard population assigned to Northern Norway, but in this case no Russian populations were actually sampled or included in the analysis. The assignment test suggested Northwest Europe (including both Russian and Norwegian populations) as source area for *S. procumbens* but Allen *et al.* (2015) found the same Svalbard specimens of *S. procumbens* to belong to the North-American/North-Atlantic group using plastid markers—the opposite group of what is reported here. One explanation for these contradictory results might be that the current population of *S. procumbens* in Svalbard was established through multiple introductions from different sources, followed by hybridization and subsequent decline in genetic variation (Allen *et al.* 2015). Multiple introductions have also been suggested for several other plant species in Svalbard (Alsos *et al.* 2007). On the other hand, the individuals from Svalbard clearly clustered with Northwest Europe (confirmed by both STRUCTURE and PCO analyses), and also showed very little genetic differentiation from other individuals within this group. An alternative explanation may therefore be that the opposing results are caused by the use of genetic markers reflecting genetic differentiation at different time scales. Plastid markers can often be more conservative than nuclear markers (see e.g. Eidesen *et al.* 2007), and may possibly reflect genetic differentiation from before colonization of Svalbard. This and the clear genetic split between *S. procumbens* from Europe and the North-Atlantic area/North-America should however be further investigated.

For *C. capillaris* and *K. simpliciuscula*, the split between main genetic groups can be explained by the inclusion of different subspecies. The main genetic groups of *C. capillaris* are for instance accompanied by morphological differentiation and greatly correspond to the two subspecies *C. capillaris* ssp. *capillaris* and *C. capillaris* ssp. *fuscidula* (but see comment in the methods section; Elven *et al.* 2011). Overall, our results indicate that *C. capillaris* ssp. *fuscidula*, *S. procumbens* and *C. tenellum* populations from Svalbard belong to the same genetic groups as populations from Russia and/or Norway—information that is valuable both when managing the Svalbard populations and also for long-term conservation of genetic variation at species level.

## Conclusions

In this study, we have shown that some of Svalbard’s most threatened plant species have experienced strong genetic founder- and/or bottleneck effects, likely due to climatic limitations. Their Svalbard occurrences should be considered as management units with importance for the long-term persistence of the species. At present, the species generally have small and/or few populations in Svalbard and the best management strategy would be further tracking of potential population size changes. This may also give valuable insights into plant responses to climate change.

## Sources of Funding

This study was financed by the Svalbard Environmental Protection Fund (grant number 09/25 to IGA).

## Contributions by the Authors

IGA conceived the idea, designed the project and served as project leader together with AKB. RE was the main taxonomic advisor. SB and IEBS contributed equally to this work and performed all laboratory and data analyses, as well as the drafting of the article. All authors conducted field collections and commented on the manuscript.

## Conflict of Interest Statement

None declared.

## Supplementary Material

Supplementary Data
