# Supplementary material for: Living on the edge: conservation genetics of seven thermophilous plant species in a high Arctic archipelago
Source: AoB Plants. 2017 Jan 19;9(1):plx001. doi: 10.1093/aobpla/plx001 (PMC5391696; doi:10.1093/aobpla/plx001)
Supplement: Supplementary Data [file plx001_Supp.docx]

Table S1. Red list categories for the study species in the Regional Red List for Svalbard in 2006, 2010 and 2015: CR=Critically endangered, EN=Endangered, VU=Vulnerable and NT=Near threatened.

| Species | Red list category  (Regional Red List for Svalbard) | | |
| --- | --- | --- | --- |
|  | 2006^1^ | 2010^2^ | 2015^3^ |
| *Botrychium lunaria* | CR | CR | CR |
| *Carex capillaris* ssp. *fuscidula* | CR | VU | NT |
| *Comastoma tenellum* | CR | EN | VU |
| *Kobresia simpliciuscula* ssp. *subholarctica* | EN | EN | EN |
| *Ranunculus wilanderi* | CR | EN | EN |
| *Sibbaldia procumbens* | CR | EN | EN |
| *Tofieldia pusilla* | NT | LC | NT |

^1^Kålås *et al.* 2006, ^2^Kålås *et al.* 2010, ^3^Henriksen and Hilmo 2015

Table S2. Species traits for the seven study species: Main mode of pollination (Insect/Wind/Not Applicable), assumed breeding system (Asexual/Cross-fertilization/Self-fertilization/Mixed), dispersal mode (Wind/Passive), life span (Annual/Biennial/Perennial), potential for clonal growth (Slight/None), ploidy level (2x= diploid, 4x=tetraploid)

|  | Pollination  mode | Breeding  system | Dispersal mode | Life span | Potential for clonal growth | Ploidy level |
| --- | --- | --- | --- | --- | --- | --- |
| *Botrychium lunaria* | NA^1^ | S^3^ | W | P | Slight | 2x^5^ |
| *Carex capillaris* | W | C | P | P | None | ? |
| *Comastoma tenellum* | I | S | P | A/B | None | 2x |
| *Kobresia simpliciuscula* | W | C | P | P | None | 4x^6^ |
| *Ranunculus wilanderi* | I^2^ | A^4^ | P | P | ? | 4x |
| *Sibbaldia procumbens* | I | M | P | P | Slight | 2x |
| *Tofieldia pusilla* | I | M | P | P | Slight | 2x |

^1^pteridophyte (Alsos *et al.* 2016a), ^2^pseudogamous (Asker and Jerling 1992, Alsos *et al.* 2016a), ^3^intragametophytic self-fertilization (Stensvold 2008), ^4^apomictic (Jonsell 2001, Alsos *et al.* 2016a), ^5^probably paleopolyploid, ^6^uncertain (Seeber *et al.* 2014)

Table S3. AFLP details: Number of samples analysed in study species and outgroups, number of primer combinations tested, primer combination used (EcoRI and MseI), number of AFLP markers obtained (excluding and including outgroup), number and percentage of polymorphic AFLP markers obtained, and “mismatch error rate” (Bonin *et al.* 2004). For *Comastoma* *tenellum*, we used the same primer combinations as Schönswetter *et al.* (2004).

| Species | Samples  [n] | Primer tests  [n] | EcoRI | MseI | Markers  [n] | Polymorphic markers  [n] | Error rate  [%] |
| --- | --- | --- | --- | --- | --- | --- | --- |
| *Botrychium lunaria*  [+ Outgroup] | 111  [+5] | 48 | FAM-AAG  PET-AGA  VIC-ACG  NED-ACA | CGA  CGA  CAC  CTA | 71  [78] | 55  77.5 % | 2.0 |
| *Carex capillaris*  [+ Outgroup] | 68  [+8] | 8 | FAM-ACT  FAM-ACC  VIC-AGG  PET-AGA | CTT  CTA  CA  CAT | 137  [151] | 83  60.6 % | 1.9 |
| *Comastoma tenellum*  [+ Outgroup] | 81  [+5] |  | FAM-ACC  NED-AGG  VIC-ACA | CAT  CAC  CAC | 101  [123] | 84  83.2 % | 2.2 |
| *Kobresia simpliciuscula*  [+ Outgroup] | 54  [+5] | 36 | FAM-ACC  NED-ACA  VIC-AAG  PET-AGA | CAG  CTC  CGA  CGA | 66  [111] | 25  37.9 % | 0.2 |
| *Ranunculus wilanderi*  [+ Outgroup] | 19  [+5] | 36 | VIC-AAG  FAM-AGC  PET-AGA  VIC-ACG | CGA  CGG  CAC  CGA | 118  [169] | 1  0.8 % | 0.9 |
| *Sibbaldia procumbens*  [+ Outgroup] | 151  [+2] | 18 | VIC-AGG  NED-ACA  FAM-ACC  NED-ACA | CAA  CAT  CAG  CAC | 111  [145] | 62  55.9 % | 1.2 |
| *Tofieldia pusilla*  [+ Outgroups] | 145  [+12] | 18 | FAM-ACC  NED-ACA  VIC-AAG  PET-AGA | CAG  CAT  CTT  CT | 68  [127] | 32  47.1 % | 1.5 |


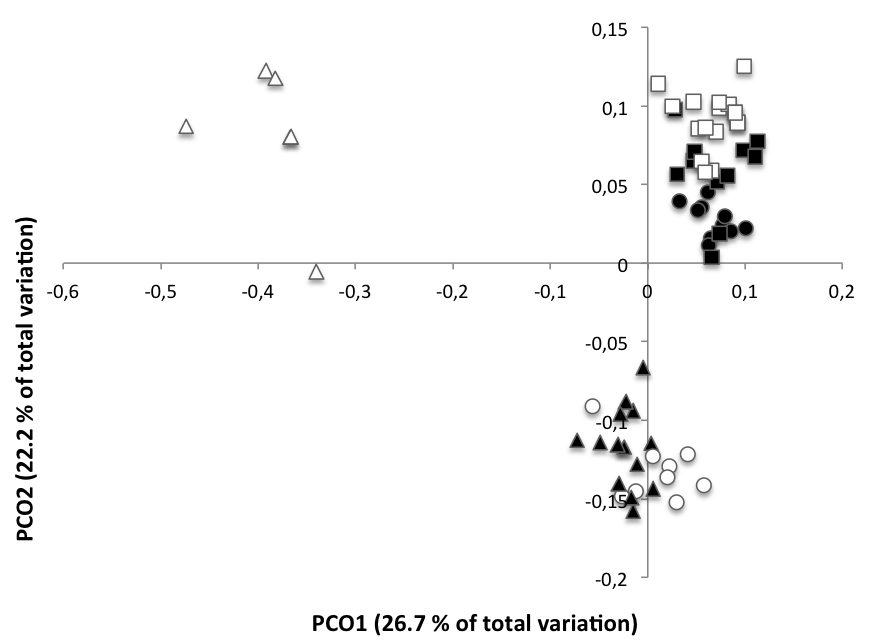


Figure S4. PCO (principal coordinates analysis) of AFLP multilocus phenotypes based on Dice similarity of 68 *Carex capillaris* individuals. The figure shows that Greenland (triangle) is separated from the other samples along the first PCO axis. Geographic regions are indicated by symbols: filled circle, Svalbard; filled square, Northern Norway; circle, Southern Norway; square, Iceland; filled triangle, Alps; triangle, Greenland.

**Table S5.** Two measures of temperature requirement, a rarity index, six measures of the genetic founder/bottleneck effects and source reference for the AFLP data for each of the species included in Figure 2. Measures of thermal requirement: (1) measured July temperature requirement in East Greenland (Karlsen and Elvebakk 2003; Temp) and (2) bioclimatic subzone where the species is frequent (f) or scattered (s) (Elven *et al.* 2011; Zone). Rarity index: relative rarity of the species quantified on a scale from 1 to 6, based on how much rarer the species is in Svalbard compared to abundances reached in its optimal habitat (Rarity). Measures of genetic founder/bottleneck effects: (1) the proportion of intrapopulation genetic diversity observed in Svalbard relative to the main source region (Population diversity), (2) the proportion of regional genetic diversity in Svalbard relative to the source region, estimated among all individuals in the region (Regional diversity), (3) the proportion of AFLP markers observed in Svalbard relative to the source region (Markers), (4) minimum number of colonizing propagules (Propagules), (5) the number of source regions estimated from the assignment test (Sources assignment), and (6) the number of source regions necessary to find all markers observed in Svalbard (Sources markers). References for the AFLP data: for *Sibbaldia procumbens* and species other than the remaining study species, the AFLP data has either been published with full information on all included measures (Alsos *et al*. 2007), published with full information on the six measures of the genetic founder effect (Alsos *et al.* 2015), or published with partial information on the measures of genetic founder effect (Westergaard *et* *al.* 2011; Gussarova *et al.* 2012).

| species | Temp. | Zone | Rarity | Population diversity | Regional diversity | Markers | Propagules | Sources assignment | Sources markers | AFLP data |
| --- | --- | --- | --- | --- | --- | --- | --- | --- | --- | --- |
| *Arabis alpina* | 5-6 °C | C (f) | 2 | 0.769 | 0.714 | 0.853 | 1 | 1 | 1 | (Alsos *et al*. 2007) |
| *Arenaria humifusa* | 5-6 °C | C (s) | 1 | 1.259 | 0.983 | 0.969 | 4 | 2 | 2 | (Westergaard *et* *al.* 2011) |
| *Betula nana* | 6-7°C | D (f) | 2 | 0.698 | 0.711 | 0.589 | 8 | 3 | 2 | (Alsos *et al*. 2007) |
| *Botrychium lunaria* | 7-8 °C | D (f) | 1 | 0.000 | 0.000 | 0.436 | 3 | 1 | 1 | This study |
| *Carex capillaris* | 6-7°C | D (f) | 1 | 0.278 | 0.224 | 0.759 | 5 | 1 | 1 | This study |
| *Cassiope tetragona* | 4-5 °C | C (f) | 4 | 0.942 | 0.965 | 0.912 | 13 | 5 | 2 | (Alsos *et al*. 2007) |
| *Comastoma tenellum* | 6-7°C | D (f) | 1 | 0.500 | 0.732 | 0.627 | 4 | 1 | 3 | This study |
| *Dryas octopetala* | 3-4 °C | C (f) | 5 | 0.837 | 0.864 | 1.398 | 30 | 4 | 6 | (Alsos *et al*. 2007) |
| *Emeptrum nigrum* | 5-6 °C | C (s) | 3 | 0.416 | 0.369 | 0.745 | 7 | 1 | 3 | (Alsos *et al*. 2007) |
| *Euphrasia wettsteinii* | 6-7°C | C (s) | 1 | 0.667 | 0.707 | 0.615 | 22 | 2 | 2 | (Gussarova *et al.* 2012) |
| *Kobresia simpliciuscula* | 6-7°C | C (s) | 1 | 0.089 | 0.304 | 0.813 | 2 | 1 | 1 | This study |
| *Rubus chamaemorus* | 6-7°C | D (f) | 1 | 0.477 | 0.523 | 0.613 | 6 | 1 | 1 | (Alsos *et al*. 2007) |
| *Sagina caespitosa* | 5-6 °C | C (s) | 1 | 0.333 | 0.247 | 0.550 | 4 | 1 | 2 | Westergaard *et* *al.* 2011 |
| *Salix herbacea* | 4-5 °C | C (s) | 3 | 0.731 | 0.745 | 0.655 | 18 | 2 | 4 | (Alsos *et al*. 2007) |
| *Saxifraga rivularis* | <3 °C | B (f) | 6 | 1.987 | 1.562 | 1.097 | 20 | 2 | 2 | (Alsos *et al*. 2007) |
| *Sibbaldia procumbens* | 6-7°C | D (f) | 1 | 0.000 | 0.000 | 0.750 | 1 | 1 | 1 | (Alsos *et al.* 2015) |
| *Tofieldia pusilla* | 6-7°C | D (f) | 2 | 0.928 | 0.872 | 0.968 | 1 | 1 | 1 | This study |
| *Vaccinium uliginosum* | 5-6 °C | C (s) | 1 | 0.382 | 0.946 | 0.779 | 12 | 2 | 3 | (Alsos *et al*. 2007) |

**Table S6.** Assignment of the Svalbard individuals of the study species to potential source areas, inferred from multi-locus assignment tests performed in AFLPOP. A log likelihood difference of one was used as a threshold for allocation. Population ID follows Table 2.

| Species | Source area (population ID) | No. of individuals assigned (%) |
| --- | --- | --- |
| *Botrychium lunaria* | Alps (Bl06; Bl07; Bl12; Bl13) | - |
|  | Faroe Islands (Bl11) | - |
|  | Greenland (Bl02) | - |
|  | Iceland (Bl03; Bl04; Bl10; Bl15) | - |
|  | Norway (Bl05; Bl08; Bl09; Bl14) | - |
| *Carex capillaris* | Alps (Cc03) | - |
|  | Greenland (Cc11) | - |
|  | Iceland (Cc02; Cc05) | - |
|  | Northern Norway (Cc06; Cc07; Cc08) | 5 (50 %) |
|  | Southern Norway (Cc09) | - |
| *Comastoma teneullum* | North-America (Ct11) | - |
|  | Alps (Ct02; Ct05; Ct09) | - |
|  | Alps/Norway (Ct06; Ct07; Ct08) | - |
|  | Russia (Ct10) | 25 (100 %) |
| *Sibbaldia procumbens* | Alps/Norway (Sp07; Sp08; Sp09; Sp11; Sp14) | - |
|  | North-America (Sp16; Sp19; Sp20) | - |
|  | North-Atlantic (Sp05; Sp06; Sp12; Sp13; Sp21) | - |
|  | Northwest Europe (Sp10; Sp17) | 25 (100 %) |
| *Tofieldia pusilla* | Greenland (Tp07) | - |
|  | Europe (Tp08-15; Tp19) | 72 (100 %) |
